# Supplementary material for: Novel Driver Strength Index highlights important cancer genes in TCGA PanCanAtlas patients
Source: PeerJ. 2022 Aug 11;10:e13860. doi: 10.7717/peerj.13860 (PMC9375969; doi:10.7717/peerj.13860)
Supplement: Supplemental Information 2 [file peerj-10-13860-s002.zip › COHORTS/genes plots/cohorts/2021_8_16_14_9_distribution_cohorts_TGCT.pdf]

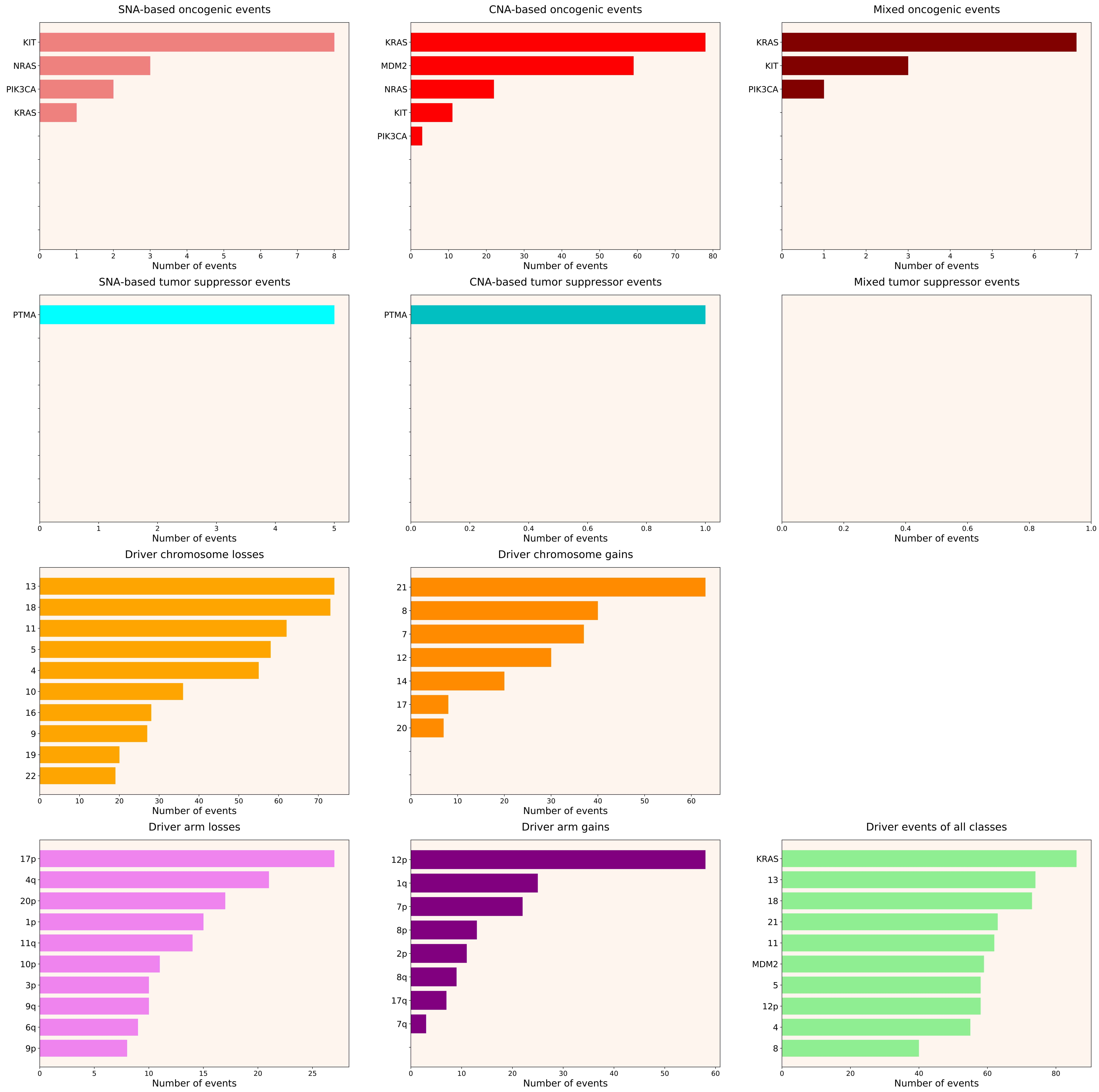

SNA-based oncogenic events
KIT
NRAS
PIK3CA
KRAS
Number of events
CNA-based oncogenic events
KRAS
MDM2
NRAS
KIT
PIK3CA
Number of events
Mixed oncogenic events
KRAS
KIT
PIK3CA
Number of events
SNA-based tumor suppressor events
PTMA
Number of events
CNA-based tumor suppressor events
PTMA
Number of events
Mixed tumor suppressor events
Number of events
Driver chromosome losses
13
18
11
5
4
10
16
9
19
22
Number of events
Driver chromosome gains
21
8
7
12
14
17
20
Number of events
Driver arm losses
17p
4q
20p
1p
11q
10p
3p
9q
6q
9p
Number of events
Driver arm gains
12p
1q
7p
8p
2p
8q
17q
7q
Number of events
Driver events of all classes
KRAS
13
18
21
11
MDM2
5
12p
4
8
Number of events
